# CRCDA—Comprehensive resources for cancer NGS data analysis

**DOI:** 10.1093/database/bav092

**Published:** 2015-10-08

**Authors:** Manonanthini Thangam, Ramesh Kumar Gopal

**Affiliations:** AU-KBC Research Centre, MIT Campus of Anna University, Chromepet, Chennai, India

## Abstract

Next generation sequencing (NGS) innovations put a compelling landmark in life science and changed the direction of research in clinical oncology with its productivity to diagnose and treat cancer. The aim of our portal comprehensive resources for cancer NGS data analysis (CRCDA) is to provide a collection of different NGS tools and pipelines under diverse classes with cancer pathways and databases and furthermore, literature information from PubMed. The literature data was constrained to 18 most common cancer types such as breast cancer, colon cancer and other cancers that exhibit in worldwide population. NGS-cancer tools for the convenience have been categorized into cancer genomics, cancer transcriptomics, cancer epigenomics, quality control and visualization. Pipelines for variant detection, quality control and data analysis were listed to provide out-of-the box solution for NGS data analysis, which may help researchers to overcome challenges in selecting and configuring individual tools for analysing exome, whole genome and transcriptome data. An extensive search page was developed that can be queried by using (i) type of data [literature, gene data and sequence read archive (SRA) data] and (ii) type of cancer (selected based on global incidence and accessibility of data). For each category of analysis, variety of tools are available and the biggest challenge is in searching and using the right tool for the right application. The objective of the work is collecting tools in each category available at various places and arranging the tools and other data in a simple and user-friendly manner for biologists and oncologists to find information easier. To the best of our knowledge, we have collected and presented a comprehensive package of most of the resources available in cancer for NGS data analysis. Given these factors, we believe that this website will be an useful resource to the NGS research community working on cancer.

**Database URL**: http://bioinfo.au-kbc.org.in/ngs/ngshome.html.

## Introduction

The chain termination method by Sanger and sequencing method by Maxam-Gilbert overturned the biomedical world through an efficient sequencing approach at significantly lower costs (1, 2). In 2004, 454 Life Sciences showcased a paralleled form of sequencing called pyrosequencing (3). The first form of their instrument decreased sequencing expenses at 6-fold contrasted with mechanized Sanger sequencing, and was the second of the new era of sequencing innovations, after massive parallel signature sequencing (4). The main difference between Sanger sequencing data and next generation sequencing (NGS) data is the read length or the quantity of nucleotides acquired. NGS is a recent innovation that empowers massively parallel sequencing reactions along these lines diminishing the specimen size and reagent costs. The sequencing process manifold to permit concurrent sequencing each reaction and to analyse the huge number of samples. Procedures in NGS include extracting DNA/RNA from samples, making a library of sections that are sequenced in parallel to short reads, and are reassembled by aligning them to a reference genome. In this way, the entire genome is obtained from the arrangement of consensus reads. NGS utilizes different platforms such as GS FLX by 454 Life Technologies/Roche, Genome Analyzer by Solexa/Illumina, SOLiD by Applied Biosystems, CGA Platform by Complete Genomics, PacBio RS by Pacific Biosciences, Polonator G.007, Ion/Proton PGM and Oxford Nanopore for sequencing genomes (5). The reads obtained from these platforms can be aligned and further analysed by using various NGS tools.

NGS experiments generate volumes of data, which requires a computationally intensive system for data storage, management and processing. The main processing feature of the system is to transform image data into sequence reads, known as base calling. On each platform, for each base in reads, image parameters such as intensity level, background and noise are utilized to generate reads and quality scores. Quality scores computed provides significant information for downstream analysis. Assembly and alignment are considered to be complicated and resource intensive steps in the NGS data analysis. The RNA data analysis also puts forward unique challenges and demands sequence alignment across spliced junctions and differential expression. In addition to that, variant calling for analysing variants, annotation for adding biological context, ChIP sequencing and methylation for analysing gene regulation are special tasks in NGS data analysis. Major applications of NGS are detecting genomic alterations and biomarkers which in turn be useful in diagnosis and treatment of cancer.

Cancer is an array of diseases defined by abnormal cell growth and is caused by mutations in somatic or germ-line cells. NGS technologies play a critical part in the diagnosis and treatment of cancer. Researchers are using NGS technologies to achieve a deeper understanding of tumor through target sequencing and to study cancer progression. Emerging methods in NGS are useful in monitoring the progression of cancer and drug response in cancer cells. Various NGS tools have been developed to analyse and interpret sequence reads and different studies have been carried out to find novel genomic variations, which cause cancer. NGS based studies through whole genome sequencing (WGS) and whole exome sequencing (WES) technologies will help us to understand the mechanism underlying progression and evolution of cancer (6).

The exponential growth of NGS data with extensive cancer studies and the development of new tools made easier for the research community to analyse NGS data. The primary bottleneck of NGS study lies in data analysis, because the complexity of NGS data analysis depends on multitude of databases, tools and heterogeneity of data involved in the study. The data analysis workflow needs to be designed carefully and tools have to be selected cautiously for structured data management and meaningful biological results. The NGS tools can be classified into commercial packages and open source tools. Commercial packages available are DNANexus, CLC Genomics Workbench and Genome Quest. Most commercial packages use proprietary algorithms for data analysis and are costlier. In contrast, researchers developed excellent software tools that may be either standalone or web-based for the analysis of progressively large genome data and made these tools open access to all.

## Methods and Resources

### NGS tools for data analysis

**Figure 1.**
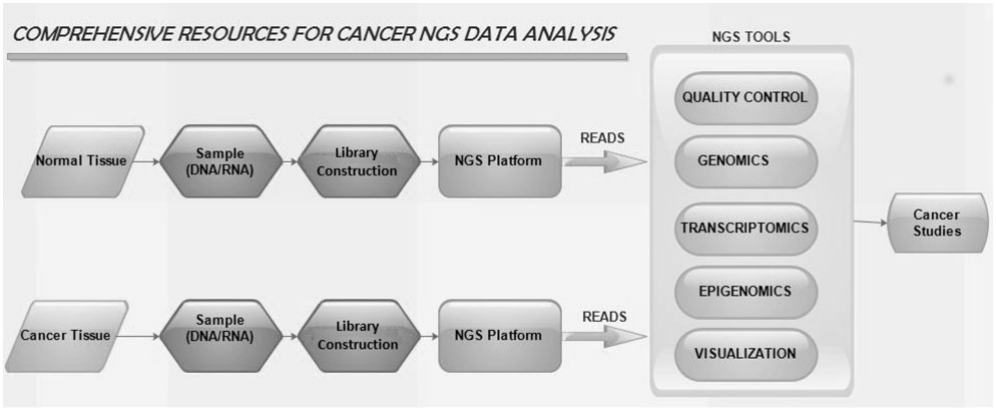
The workflow of NGS tools in cancer studies.

NGS tools used in our web portal are grouped into five categories. They are cancer genomics, cancer transcriptomics, quality control (QC), cancer epigenomics and cancer genome visualization. The workflow of NGS tools is shown in Figure 1. Different tools listed are restricted to the criteria that they are available for either online or standalone and are strictly confined to analyse NGS data.

### Cancer genomics

The term ‘Genomics’ was first coined by McKusick and Ruddle in September 1987 (7) as a name for their new journal. Genomics is a discipline that applies to analysing the structure and function of genomes. Cancer genomics denote sequencing a genome that is confined to a particular tumor tissue and then mapping the short reads obtained to a reference genome. Cancer genomics tools were clustered into four main groups such as assembly, alignment, annotation and variation discovery based on their application. Variation discovery tools under distinct subsections like mutation calling, structural variations, copy number variations (CNVs), indel and mutation effects are listed along with other cancer genomics tools for annotation, assembly and aligment in Table 1. Assembly denotes aligning and blending short reads from NGS experiment to recreate the original sequence. Alignment is a crucial step in resequencing and it refers to align short reads from NGS experiment to a reference genome. With reference genome in hand, alignment is used for detecting variants in samples. Annotation is the process of identifying the position and biological context of genes in a genome. Genome variations include mutations, polymorphisms and structural variations. The term mutation is often used to refer to a genome variation that is related to certain hereditary disease or cancer, while polymorphism indicates a modification that can be neither harmful nor beneficial (65). Various tools have been listed under genomic variation discovery. They are grouped into five subdivisions such as mutation calling, structural variations, CNVs, indel and mutation effects. Mutation calling tools are used to identify and annotate mutations in reads. Indel tools are used to find small indels, and structural variation tools are used to detect changes in chromosomal structure. CNVs are genomic differences that occur due to deletion or duplication of larger regions of DNA. Moreover, the effect of mutation on a specific site occurred by certain amino acid substitution can be predicted by using mutation effect predication tools. Even though the tools are listed under precise section, they may serve more than one purpose. For example, VarScan tool though listed under CNV, also detect indels.

**Table 1.** List of tools for cancer genomics

Category	Tool	URL	Reference
Alignment	BFAST	http://sourceforge.net/apps/mediawiki/bfast/	(8)
	BWA	http://bio-bwa.sourceforge.net/	(9, 10)
	Bowtie	http://bowtie-bio.sourceforge.net/index.shtml	(11)
	NovoalignCS	http://www.novocraft.com/Novoalign/	
	MAQ	http://maq.sourceforge.net/	(12)
	SHRiMP	http://compbio.cs.toronto.edu/shrimp/	(13)
	SOAP2	http://soap.genomics.org.cn/	(14)
	SSAHA2	http://www.sanger.ac.uk/resources/software/ssaha2/	(15)
	GASSST	http://www.irisa.fr/symbiose/projects/gassst/	(16)
	PASS	http://pass.cribi.unipd.it/	(17)
	MicroRazerS	http://www.seqan.de/projects/MicroRazerS/	(18)
	SeqMap	http://www-personal.umich.edu/∼jianghui/seqmap/	(19)
	PerM	http://code.google.com/p/perm/	(20)
Assembly	ALLPATHS-LG	http://broadinstitute.org/software/allpaths-lg/blog/?page_id=12	(21, 22)
	Celera Assembler	http://wgs-assembler.sourceforge.net/wiki/index.php?title=Main_Page	(23)
	Geneious	http://www.geneious.com/workflows/genomics	
	LOCAS	http://locas.sourceforge.net	(24)
	Contrail	http://sourceforge.net/projects/contrail-bio/	
	MIRA	http://sourceforge.net/p/mira-assembler/wiki/Home	(25)
	Velvet	http://www.molecularevolution.org/software/genomics/velvet/	(26)
	CongrPE	http://sourceforge.net/projects/congrpe	
	ZORRO	http://lge.ibi.unicamp.br/zorro	
	ABySS	http://bcgsc.ca/platform/bioinfo/software/abyss	(27)
Annotation	wANNOVAR	http://wannovar.usc.edu/	(28)
	ANNOVAR	http://www.openbioinformatics.org/annovar/	(29)
	SVA	http://www.svaproject.org/download.php	(30)
	WebApollo	http://gmod.org/wiki/WebApollo	(31)
	CHAoS	http://www.well.ox.ac.uk/∼kgaulton/chaos.shtml	
	COVA	https://sourceforge.net/p/cova/wiki/Home/	
Genomic variation discovery	GAMES	http://aqua.unife.it/GAMES/	(32)
	CoNAn-SNV	http://omictools.com/sequencing/genome-resequencing/germline-calling/conan-snv-s532.html	(33)
	LoFreq	http://sourceforge.net/projects/lofreq/	(34)
	Unified_genotyper GATK	http://www.broadinstitute.org/gsa/wiki/index.php/	(35)
	JointSNVMix	http://compbio.bccrc.ca	(36)
	SAMtools	http://samtools.sourceforge.net/	(37)
	SNVMix	http://compbio.bccrc.ca/?page_id=204	(38)
	Strelka	https://sites.google.com/site/strelkasomaticvariantcaller/	(39)
	SOAPsnp	http://soap.genomics.org.cn/soapsnp.html	
	SomaticSniper	http://genome.wustl.edu/software/somaticsniper	(40)
	VarScan	http://varscan.sourceforge.net/	(41)
	Dindel	http://www.sanger.ac.uk/resources/software/dindel/	(42)
	Pindel	https://trac.nbic.nl/pindel/	(43)
	SplazerS	http://www.seqan.de/projects/	
	MoDIL	http://compbio.cs.toronto.edu/modil/	(44)
	PyroHMMvar	https://code.google.com/p/pyrohmmvar/	(45)
	MuTect	https://www.broadinstitute.org/cancer/cga/mutect	
Structural variation	SVseq2	http://www.engr.uconn.edu/∼jiz08001/svseq2.html	(46)
	BreakDancer	http://breakdancer.sourceforge.net/	(47)
	CREST	ftp://ftp.stjude.org/pub/software/CREST/CREST.tgz	
	GASV	http://code.google.com/p/gasv/	
	HYDRA	http://code.google.com/p/hydra-sv/	(48)
	PEMer	http://sv.gersteinlab.org/pemer/	(49)
	R453Plus1Toolbox	http://www.bioconductor.org/packages/2.10/bioc/html/R453Plus1Toolbox.html	(50)
	SVMerge	http://svmerge.sourceforge.net/	(51)
	SVDetect	http://svdetect.sourceforge.net/Site/Home.html	(52)
	VariationHunter	http://compbio.cs.sfu.ca/strvar.htm	(53)
	deStruct	https://code.google.com/p/destruct/	
CNV	CMDS	https://dsgweb.wustl.edu/qunyuan/software/cmds/	(54)
	CBS	https://r-forge.r-project.org/R/?group_id=702	
	CNAseg	http://www.compbio.group.cam.ac.uk/Resources/CNAseg/CNAseg.rar	(55)
	cnvHMM	http://genome.wustl.edu/software/cnvhmm	
	CNVnator	http://sv.gersteinlab.org/cnvnator/	(56)
	FREEC	http://bioinfo-out.curie.fr/projects/freec/	(57)
	RDXplorer	http://sourceforge.net/projects/rdxplorer/	(58)
	SegSeq	http://www.broadinstitute.org/cgi-bin/cancer/publications/pub_paper.cgi?mode=view&paper_id=182	(59)
	VarScan	http://varscan.sourceforge.net/	(41)
	GENSENG	http://sourceforge.net/projects/genseng/	
	CNV-seq	http://tiger.dbs.nus.edu.sg/cnv-seq/	(60)
	mrCaNaVaR	http://mrcanavar.sourceforge.net/	(61)
	Onco SNP-SEQ	https://sites.google.com/site/oncosnpseq/	
	Control-FREEC	http://bioinfo-out.curie.fr/projects/freec/	
	BIC-seq	http://compbio.med.harvard.edu/Supplements/PNAS11.html	
Mutation effect	ANNOVAR	http://www.openbioinformatics.org/annovar/	
	PolyPhen-2	http://genetics.bwh.harvard.edu/pph2/	(62)
	CHASM	http://wiki.chasmsoftware.org	(63)
	SIFT	http://sift.jcvi.org/	(64)

### Cancer transcriptomics

Transcriptomics is the study of complete RNA transcript (transcriptome) produced by a genome at specific conditions. NGS technologies are applied to study cDNA fragments to deliver a transcriptional profile. Transcriptomics involves alignment and analysis of RNA sequence reads and they are aligned using RNA specific aligners to detect new splicing junctions. Differential expression tools are used to quantify the expression values of reads. Gene fusion tools are used to align reads comprising fusion junctions to the genome. Technical improvements and decreasing expenses made transcriptome analysis a routine in cancer research and it provides boundless potential in cancer research. The transcriptomic tools are classified as spliced alignment, differential expression, alternative splicing and gene fusion and are listed in Table 2. They are used to understand how transcripts are altered by diseases such as cancer and how these altered transcripts play a significant role in distinguishing cancer and its subtypes (88).

**Table 2.** List of tools for cancer transcriptomics

Category	Tool	URL	Reference
Spliced alignment	TopHat	http://tophat.cbcb.umd.edu/	(66)
	QPALMA	http://www.fml.mpg.de/raetsch/projects/qpalma	(67)
	MapSplice	http://www.netlab.uky.edu/p/bioinfo/MapSplice	(68)
	SpliceMap	http://www.stanford.edu/group/wonglab/SpliceMap/	(69)
	GMAP	http://research-pub.gene.com/gmap/	
	STAR	http://gingeraslab.cshl.edu/STAR/	(70)
	SOAPsplice	http://soap.genomics.org.cn/soapsplice.html	(71)
	Supersplat	http://mocklerlab.org/tools/1	(72)
Differential expression	EdgeR	http://www.bioconductor.org/packages/2.11/bioc/html/edgeR.html	(73)
	CuffDiff	http://cufflinks.cbcb.umd.edu/	(74)
	DESeq	http://www-huber.embl.de/users/anders/DESeq/	(75)
	Myrna	http://bowtie-bio.sourceforge.net/myrna/index.shtml	(76)
Alternative splicing	CuffDiff	http://cole-trapnell-lab.github.io/cufflinks/cuffdiff/	(74)
	MISO	http://genes.mit.edu/burgelab/miso/	(77)
	DEXseq	http://bioconductor.org/packages/release/bioc/html/DEXSeq.html	(78)
	ALEXA-Seq	http://www.alexaplatform.org/alexa_seq/	(79)
	SOAPdenovo-Trans	http://sourceforge.net/projects/soapdenovotrans/	(80)
Gene fusion	Defuse	http://sourceforge.net/apps/mediawiki/defuse/index.php?title=Main_Page	(81)
	FusionAnalyser	http://www.ilte-cml.org/FusionAnalyser/	(82)
	FusionHunter	http://bioen-compbio.bioen.illinois.edu/FusionHunter/	(83)
	FusionMap	http://www.omicsoft.com/fusionmap/	(84)
	FusionSeq	http://archive.gersteinlab.org/proj/rnaseq/fusionseq/	(85)
	SOAPfusion	http://soap.genomics.org.cn/SOAPfusion.html	(86)
	TopHat-Fusion	http://ccb.jhu.edu/software/tophat/index.shtml	(87)

### Quality control

QC is the first step in the NGS data analysis after getting raw sequence reads from next generation sequencers. In NGS experiments, shorter reads obtained may contain erroneous data like poor quality reads, adapter sequences, base calling errors and some insertions/deletions among the original reads. Definite screening techniques and filtration criteria like sequence quality, sequence length, etc. are used to minimize errors in sequence reads (89). In addition to these methods, certain software tools are used to detect contaminated and low quality reads called QC tools. QC tools use different algorithms to detect and filter artifacts in reads obtained from NGS methods. The error detection and correction tools for QC are listed in Table 3. The reads obtained after QC are further filtered for primer contamination to improve and ensure quality of reads. Read quality has to be checked carefully before initiating NGS data analysis because there is no utility present in downstream analysis tools to remove erroneous data in reads. In short, the quality of the output depends on the quality of the input in terms of quality reads.

**Table 3.** List of tools for QC

Category	Tool	URL	Reference
Error detection and correction	NGSQC Toolkit	www.nipgr.res.in/ngsqctoolkit.html	(89)
	SHREC	http://shrec-ec.sourceforge.net/	(90)
	TagDust	http://tagdust.sourceforge.net/	(91)
	AYB	http://www.ebi.ac.uk/goldman-srv/AYB/	
	BayesCall	http://www.cs.berkeley.edu/∼yss/bayescall/	(92)
	Ibis	https://bioinf.eva.mpg.de/Ibis/	(93)
	Swift	http://sourceforge.net/projects/swiftng/	(94)
	QuorUM	http://www.genome.umd.edu/quorum.html	
	HiTEC	http://www.csd.uwo.ca/∼ilie/HiTEC/	(95)
	Musket	http://musket.sourceforge.net/homepage.htm#latest	(96)
	ECHO	http://uc-echo.sourceforge.net/	(97)
	Trowel	http://sourceforge.net/projects/trowel-ec/	(98)
	Reptile	http://aluru-sun.ece.iastate.edu/doku.php?id=reptile	(99)
	HECTOR	http://sourceforge.net/projects/hector454/	(100)
	DecGPU	http://decgpu.sourceforge.net/homepage.htm#latest	(101)
	Hybrid SHREC	http://www.cs.helsinki.fi/u/lmsalmel/hybrid-shrec/	
	HTQC	http://sourceforge.net/projects/htqc/	(102)
	QC-Chain	http://www.computationalbioenergy.org/qc-chain.html	(103)
	Kraken	http://www.ebi.ac.uk/research/enright/software/kraken	(104)

### Cancer epigenomics

Many cancers involve multiple factors like environmental factors or genetic factors with impact on interlinked biological pathways and the environmental effects are mediated through epigenetic modifications. The study of epigenetic changes that occur on a genome is referred as epigenomics. The advent of NGS, has empowered significant progress in the study of triggering, and progression of cancer. Epigenetic changes such as DNA methylation, modification of histones and miRNA silencing are also responsible for cancer. However, they do not produce any nucleotide change in the sequence. DNA methylation, histone modifications and furthermore, miRNA silencing play a major role in gene regulation. Sometimes, loss of methylation at general methylated sites (hypomethylation) and gain of methylation at the abnormal sites (hypermethylation) lead to cancer (105). ChIP Seq tools are used to discover motifs and identify histone modifications from enriched domains and peak regions. Epigenetic changes in a genome have the potential to explain complex disease mechanisms. In particular, DNA methylation plays a major role in genome evolution and histone modification. Methylation tools are used to generate methylation maps for analysis. Different available tools for cancer epigenomics are classified as Methylation, ChIP Seq and Bisulphite Seq, and they are listed in Table 4.

**Table 4.** List of tools for cancer epigenomics

Category	Tool	URL	Reference
ChIP Seq	MACS	http://liulab.dfci.harvard.edu/	(106)
	PeakSeq	http://info.gersteinlab.org/PeakSeq	(107)
	S-Mart	https://urgi.versailles.inra.fr/Tools/S-Mart	(108)
	SICER	http://home.gwu.edu/∼wpeng/Software.htm	(109)
	MEME-ChIP	http://meme.nbcr.net/meme/cgi-bin/meme-chip.cgi	(110)
	GEM	http://cgs.csail.mit.edu/onePageGem/	(111)
	DREME	http://meme.nbcr.net/meme/doc/dreme.html	(112)
Bisulphite Seq	Bis-SNP	http://epigenome.usc.edu/publicationdata/bissnp2011/	(113)
	bsmap	https://code.google.com/p/bsmap	(114)
	BRAT	http://compbio.cs.ucr.edu/brat/	(115)
	BatMeth	http://code.google.com/p/batmeth/	
	B-SOLANA	http://code.google.com/p/bsolana	(116)
	PASS-bis	http://pass.cribi.unipd.it/cgi-bin/pass.pl?action=Download	(117)
	Bismark	http://www.bioinformatics.babraham.ac.uk/projects/bismark/	(118)
	Kismeth	http://katahdin.mssm.edu/kismeth/revpage.pl	(119)
	BS Seeker	http://pellegrini.mcdb.ucla.edu/BS_Seeker/BS_Seeker.html	(120)
Methylation	NGSmethPipe	http://bioinfo2.ugr.es/NGSmethPipe/	
	bsmooth-align	https://github.com/BenLangmead/bsmooth-align	
	methylkit	https://code.google.com/p/methylkit/	(121)
	methylumi	http://www.bioconductor.org/packages/release/bioc/html/methylumi.html	
	methylcode	https://github.com/brentp/methylcode	(122)

### Cancer genome visualization

The alignment and assembly data can be examined by using graphical tools for analysing the output files such as FASTQ, SAM (Sequence Alignment Map format), BAM (Binary compressed SAM format), VCF (Variant Call Format), etc. from various NGS tools. Genome visualization tools provide an interface to visualize data, results and annotations associated with a particular genome of interest. Annotation data, genetic information, transcripts pattern, etc. are provided along with the genomic data. The visualization tool can either be a standalone tool that can be installed on a local computer or a web browser tool. Most visualization tools are provided with a graphical user interface (GUI) so that user can view data or results, edit data, color and zoom. In some tools, search operations can also be performed. Visualization tools for data visualization with data interpretation are listed in Table 5.

**Table 5.** List of tools for visualization

Category	Tool	URL	Reference
Visualization	Strand NGS	http://www.strand-ngs.com/	
	CIRCOS	http://circos.ca/	(123)
	IGV	http://www.broadinstitute.org/igv/	(124, 125)
	Tablet	http://ics.hutton.ac.uk/tablet	(126)
	BamView	http://bamview.sourceforge.net/	(127, 128)
	EagleView	http://bioinformatics.bc.edu/marthlab/wiki/index.php/EagleView	(129)
	NGSView	http://ngsview.sourceforge.net/	(130)
	ZOOM Lite	http://bioinfor.com/zoom/lite	(131)
	UCSC Genome Browser	http://genome.ucsc.edu/	(132)
	Genplay	http://genplay.einstein.yu.edu/wiki/index.php/Main_Page	(133, 134)
	Savant	http://genomesavant.com/p/savant/index	
	ABrowse	http://www.abrowse.org/	(135)
	Integrated Genomic Browser	http://bioviz.org/igb	
	Artemis	http://www.sanger.ac.uk/resources/software/artemis	(136, 137)

### NGS pipeline tools

Many tools are available for NGS data analysis, yet their use often limited to skilled bioinformaticians since these tools have been developed in different programming languages for different operating systems. For instance, Bowtie is an excellent tool for aligning sequencing reads but will be complicated for biologists to install, configure and use. To overcome the difficulty of individual tool developers designed certain workflows called pipelines. Managing NGS reads, handling and configuring NGS tools are difficult tasks for biologists and biotechnologists who work on NGS data. NGS Pipelines, a collection of structured commands or software tools specific to a particular platform or data are used to improve productivity and specificity of data processing. Pipelines can be either general (for data analysis) or specific (for QC and variation calling). Pipelines implement simple user interface, and most of the tools are cross platform (138). Variant calling pipeline tools are used to detect aberrations, polymorphisms and indels. Variant calling pipeline tools, QC pipelines and data analysis pipelines are listed in Table 6. Recent development in pipelines and protocols permit researchers to overcome the technical issues related to handling NGS tools. For instance, in Galaxy webserver (https://usegalaxy.org/), pipelines are referred as customized workflows which include more than one Galaxy tool in sequential form for automated running of tools. Another example of pipeline is DDBJ read annotation pipeline, which is a cloud based pipeline for annotation of NGS data reads. The DDBJ Pipeline offers a GUI for processing NGS datasets using decentralized processing by NIG supercomputers currently at free of cost (140). The success of NGS data analysis lies in the selection of NGS pipeline specific to particular NGS platform and organism of study.

**Table 6.** List of pipelines

Category	Tool	URL	Reference
QC pipelines	QC-Chain	http://www.computationalbioenergy.org/qc-chain.html	
	NGSClean	https://github.com/fgvieira/ngsClean	
	NGSQC Pipeline	http://brainarray.mbni.med.umich.edu/brainarray/ngsqc/	(139)
Data analysis	HiPipe	http://hipipe.ncgm.sinica.edu.tw/	
	Galaxy	https://usegalaxy.org/	
	DDBJ Pipeline	http://p.ddbj.nig.ac.jp/	(140)
	ngs_backbone	http://bioinf.comav.upv.es/ngs_backbone/	
	NARWHAL	https://trac.nbic.nl/narwhal	
	ASAP	http://biostat.mc.vanderbilt.edu/wiki/Main/ASAP	(141)
	BreakFusion	http://bioinformatics.mdanderson.org/main/BreakFusion	(142)
	ChAMP	http://www.bioconductor.org/packages/2.13/bioc/html/ChAMP.html	(143)
	SMASHCommunity	http://www.bork.embl.de/software/smash/	(144)
	A5	http://code.google.com/p/ngopt/wiki/A5PipelineREADME	
	iMetAMOS	http://omictools.com/sequencing/de-novo-genome-sequencing/genome-assemblers/imetamos-s5034.html	(145)
	QUASR	http://quasr.sourceforge.net/	
	RUM	http://cbil.upenn.edu/RUM/	(146)
	SHORE	http://omictools.com/common-tools/analytical-pipelines/shore-s521.html	
Variant calling	cn.mops	http://bioconductor.org/packages/release/bioc/html/cn.mops.html	(147)
	inGAP-sv	http://ingap.sourceforge.net/	
	bcbio-nextgen	https://bcbio-nextgen.readthedocs.org/en/latest/contents/pipelines.html	
	MSG	http://genomics.princeton.edu/AndolfattoLab/MSG.html	(148)
	Speedseq	https://github.com/cc2qe/speedseq	
	ASAP	http://biostat.mc.vanderbilt.edu/wiki/Main/ASAP	

### NGS file converters

Most common file formats related to NGS data analysis are FASTA, FASTQ, QSEQ, SFF (Standard Flowgram Format), SAM, BAM, VCF, BED (Browser Extensible Data format), etc. Most NGS sequence files are in FASTQ or FASTA formats, which incorporate reads and quality scores. If sequence reads are mapped to the reference sequence, we get either SAM or BAM file format as output files. Sometimes it might be vital to convert one file format to another for data analysis. For instance, VCF with gene sequence variation information is no longer maintained by the 1000 Genomes Project (http://www.1000genomes.org/) and QSEQ files are plain text files generated by earlier Illumina machines. So, we need to convert these file formats into commonly used file formats like FASTQ for analysis (149, 150). The tools used for NGS file format conversion are listed in Table 7.

**Table 7.** List of tools for file format conversion

Category	Tool	URL	Reference
File converters	SRA Toolkit	http://www.ncbi.nlm.nih.gov/Traces/sra/sra.cgi?cmd=show&f=software&m=software&s=software	
	FASTX-Toolkit	http://hannonlab.cshl.edu/fastx_toolkit/	
	NGSQC Toolkit	http://www.nipgr.res.in/ngsqctoolkit.html	(89)
	Picard	http://broadinstitute.github.io/picard/	
	Bamtools	https://github.com/pezmaster31/bamtools	
	SAMtools	http://samtools.sourceforge.net/	
	GenePattern	http://www.broadinstitute.org/cancer/software/genepattern/modules?taskType=Data+Format+Conversion	
	PRINSEQ	http://prinseq.sourceforge.net/	
	PGDSpider	http://www.cmpg.unibe.ch/software/PGDSpider/	
	Galaxy	https://usegalaxy.org/	

### Cancer resources

Cancer resources, although not mainly useful to individual patients, are essential for healthcare professionals and researchers to develop strategies that can tackle challenges posed by cancer. Among the resources available for cancer, The Cancer Genome Atlas (TCGA) Data Portal furnishes an important platform for researchers to download, and analyse data sets generated by TCGA (151). Cancer resources section contains four different types of data which might be useful to any researcher working with cancer. They are (i) Cancer study data, list of articles clustered under different cancer types. (ii) Cancer Databases, list of cancer databases and oncogenomic browsers available. (iii) Cancer projects, list of ongoing projects in cancer. (iv) Cancer Pathways, list of cancer pathways. Meta analysis is a statistical analysis that is connected to comparative experiments of different and independent researchers that includes pooling the data and utilizing the pooled information to test the effectiveness of the study (152). In cancer data resources, literature data have been collected and included in the list only if the study was on cancer oriented in human and method of sequencing used must be NGS and also the literature must be published in peer-reviewed journals. The collected list of literature is displayed in the form of a list. Under cancer databases section, browsers and databases listed provide cancer related information like oncogenes, suppressor genes, methylation data and mutation data. In cancer projects section, different cancer projects by research centers and Institutes like Wellcome Trust (http://www.sanger.ac.uk/research/projects/cancergenome/), International Cancer Genome Consortium (https://icgc.org/icgc), etc. are incorporated to understand the molecular basis of cancer and gene expression profiles of different cancer types at different stages. In Cancer Pathways section, interactive pathway maps of different types of cancer from KEGG PATHWAY (www.genome.jp/kegg/) database are listed. The interactive pathway map helps us to understand interrelated oncogenes for each cancer listed.

### Web page development

The web pages were developed using hyper text markup language (HTML) language and cascaded style sheets (CSSs) for consistent styling with hyperlinks to various tools, literature, databases, pathways and projects.

### Database construction

The 1000 Genomes Project was the first multi-terabytes submitter to two sequence read archives (SRAs), the European nucleotide archive (ENA) SRA and the NCBI SRA (153). SRA data from NGS platforms make sequence data access to researchers to enhance reproducibility and novel discoveries by analysing data sets. The literature data and SRA data extracted from the NCBI SRA (http://www.ncbi.nlm.nih.gov/sra/) were stored using MySQL (http://www.mysql.com/), an extensively used open source relational database management system for biological research. The literature data collected from NCBI PubMed (http://www.ncbi.nlm.nih.gov/pubmed/), were annotated with gene data so that the literature search could be done based on either cancer or gene. The literature data include all primary citation details like Author, Title, PubMed ID (identifier), Cancer type and Journal Details. Literature and gene data include gene id in addition to all primary citation details. SRA data listed in the table consist of experiment accession, study accession, title, submitter, technology, library source and library selection.

### Search page implementation

**Figure 2.**
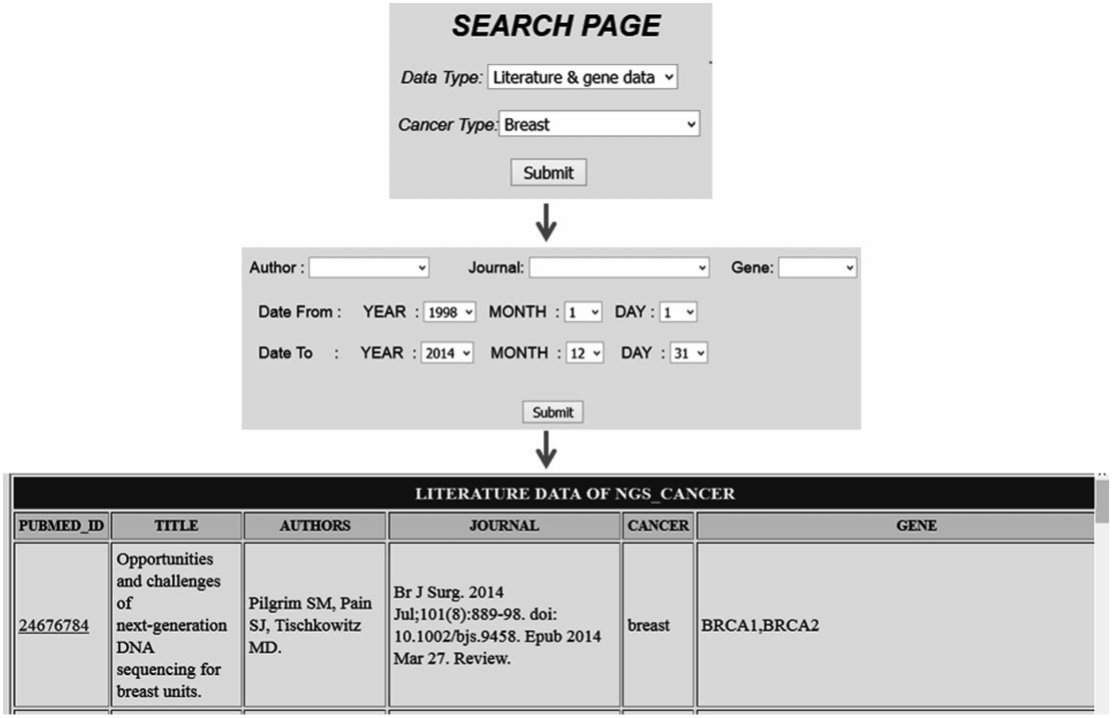
Search page accessed based on cancer type which lists all citation details with gene data for a particular cancer type.

**Figure 3.**
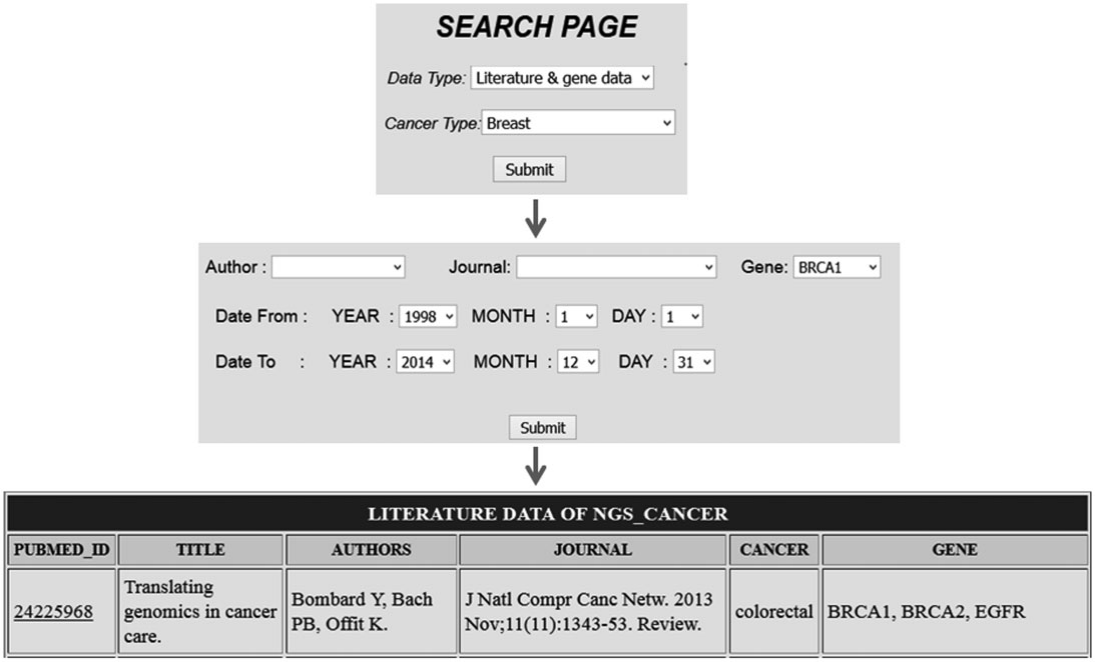
Search based on gene name which list all citation details for a particular gene in all cancer types.

Comprehensive resources for cancer NGS data analysis (CRCDA) can be queried based on (i) type of data and (ii) type of cancer. The data available for search is of three types, (i) literature and gene data, (ii) literature data and (iii) SRA data. The literature and SRA data can be queried using the search page and the search scripts were coded using PHP, a widely used scripting language. The literature database contains articles related to major cancer types such as lung, liver, breast, colorectal, prostate, gastric, cervix, bladder, non-Hodgkin lymphoma, leukemia, pancreas, kidney, endometrial, oral, thyroid, brain, ovary and skin cancers. Cancer types were selected based on their abundant existence in world population. SRA data for certain cancer types like esophagal and prostate cancer was not available at the time of database construction. So, SRA data for these types of cancer will be uploaded into the database later. The literature data can be queried either based on cancer type or gene name. The search page for literature can be accessed in two ways as shown in the following Figures 2 and 3. For example, in Figure 2 the literature data for breast cancer can be searched by selecting ‘breast cancer’ in cancer type from dropdown menu (Figure 2) and in Figure 3 the literature data for gene ‘BRCA1’ can be selected by selecting BRCA1 from gene dropdown menu. The literature data listed in gene data include all cancer types which involve BRCA1 (Figure 3). The literature data were listed as default from January 1995 to December 2014. So, user can select data from any time period within this specified limit, and the user can also search the database using the first author’s name and journal details.

## Conclusion

The main application of NGS technology through WES and WGS in cancer research has made researchers to understand the molecular landscape of different types of cancer. CRCDA is the first web portal which provides literature, tools, pipelines, pathway and SRA specific to NGS and cancer. Here, we have listed nearly 180 and above software tools in the portal under tools and pipelines and more than 500 publication information of NGS studies, which would be useful for researchers working in oncology. Peer-reviewed articles on NGS-cancer studies, cancer databases, cancer pathway data would also be beneficial to enrich cancer research in a more efficient way. Availability of all cancers and NGS-related information in one portal provides very easy and quick reference for oncology researchers.

## Future Work

Future plans include updating tools and literature data once in every 6 months to remove outdated tools and to update literature data in the database. A search page has been planned to search tools under each category and a rating option to help users to select and use most rated best tools. Public data mining tools will also be incorporated to enhance the value of this database. 

## References

[1] Sanger F. Nicklen S. Coulson A.R. (1977) DNA sequencing with chain-terminating inhibitors. Proc. Natl. Acad. Sci. U.S.A., 74, 5463–5467. 271968 10.1073/pnas.74.12.5463 PMC431765

[2] Maxam A.M. Gilbert W. (1977) A new method for sequencing DNA. Proc. Natl. Acad. Sci. U.S.A., 74, 560–564. 265521 10.1073/pnas.74.2.560 PMC392330

[3] Stein R.A. (2008) Next-generation sequencing update. Genet. Eng. Biotechnol. News, 28.

[4] Schuster S.C. (2008) Next-generation sequencing transforms today’s biology. Nat. Methods, 5, 16–18. 18165802 10.1038/nmeth1156

[5] Bodi K. (2011) Tools for next generation sequencing data analysis. J. Biomol. Tech., 22, S18.

[6] Pabinger S. Dander A. Fischer M. (2014) A survey of tools for variant analysis of next-generation genome sequencing data. Brief. Bioinform., 15, 256–278. 23341494 10.1093/bib/bbs086 PMC3956068

[7] McKusick V.A. Ruddle F.H. (1987) Toward a complete map of the human genome. Genomics, 1, 103–106. 3480265 10.1016/0888-7543(87)90001-2

[8] Homer N. Merriman B. Nelson S.F. (2009) BFAST: an alignment tool for large scale genome resequencing. PLoS One, 4, e7767. 19907642 10.1371/journal.pone.0007767 PMC2770639

[9] Li H. Durbin R. (2009) Fast and accurate short read alignment with Burrows-Wheeler transform. Bioinformatics, 25, 1754–1760. 19451168 10.1093/bioinformatics/btp324 PMC2705234

[10] Li H. Durbin R. (2010) Fast and accurate long-read alignment with Burrows-Wheeler transform. Bioinformatics, 26, 589–595. 20080505 10.1093/bioinformatics/btp698 PMC2828108

[11] Langmead B. Salzberg S.L. (2012) Fast gapped-read alignment with Bowtie 2. Nat. Methods, 9, 357–359. 22388286 10.1038/nmeth.1923 PMC3322381

[12] Li H. Ruan J. Durbin R. (2008) Mapping short DNA sequencing reads and calling variants using mapping quality scores. Genome Res., 18, 1851–1858. 18714091 10.1101/gr.078212.108 PMC2577856

[13] Rumble S.M. Lacroute P. Dalca A.V. (2009) SHRiMP: accurate mapping of short color-space reads. PLoS Comput. Biol., 5, e1000386. 19461883 10.1371/journal.pcbi.1000386 PMC2678294

[14] Li R. Yu C. Li Y. (2009) SOAP2: an improved ultrafast tool for short read alignment. Bioinformatics, 25, 1966–1967. 19497933 10.1093/bioinformatics/btp336

[15] Ning Z. Cox A.J. Mullikin J.C. (2001) SSAHA: a fast search method for large DNA databases. Genome Res., 11, 1725–1729. 11591649 10.1101/gr.194201 PMC311141

[16] Rizk G. Lavenier D. (2010) GASSST: global alignment short sequence search tool. Bioinformatics, 26, 2534–2540. 20739310 10.1093/bioinformatics/btq485 PMC2951093

[17] Campagna D. Albiero A. Bilardi A. (2009) PASS: a program to align short sequences. Bioinformatics, 25, 967–968. 19218350 10.1093/bioinformatics/btp087

[18] Emde A.K. Grunert M. Weese D. (2010) MicroRazerS: rapid alignment of small RNA reads. Bioinformatics, 26, 123–124. 19880369 10.1093/bioinformatics/btp601

[19] Jiang H. Wong W.H. (2008) SeqMap: mapping massive amount of oligonucleotides to the genome. Bioinformatics, 24, 2395–2396. 18697769 10.1093/bioinformatics/btn429 PMC2562015

[20] Chen Y. Souaiaia T. Chen T. (2009) PerM: efficient mapping of short sequencing reads with periodic full sensitive spaced seeds. Bioinformatics, 25, 2514–2521. 19675096 10.1093/bioinformatics/btp486 PMC2752623

[21] Gnerre S. MacCallum I. Przybylski D. (2011) High-quality draft assemblies of mammalian genomes from massively parallel sequence data. Proc. Natl. Acad. Sci. U.S.A., 4, 1513–1518. 21187386 10.1073/pnas.1017351108 PMC3029755

[22] Ribeiro F. Przybylski D. Yin S. (2012) Finished bacterial genomes from shotgun sequence data. Genome Res., 22, 2270–2277. 22829535 10.1101/gr.141515.112 PMC3483556

[23] Denisov G. Walenz B. Aaron L. (2008) Consensus generation and variant detection by Celera Assembler. Bioinformatics, 24, 1035–1040. 18321888 10.1093/bioinformatics/btn074

[24] Klein J.D. Ossowski S. Schneeberger K. (2011) LOCAS - a low coverage assembly tool for resequencing projects. PLoS One., 6, e23455. 21858125 10.1371/journal.pone.0023455 PMC3156226

[25] Chevreux B. Pfisterer T. Drescher B. (2004) Using the mira EST assembler for reliable and automated mRNA transcript assembly and SNP detection in sequenced EST, Genome Res., 14, 1147–1159. 15140833 10.1101/gr.1917404 PMC419793

[26] Zerbino D.R. McEwen G.K. Margulies E.H. (2009) Pebble and rock band: heuristic resolution of repeats and scaffolding in the velvet short - read de novo assembler. PLoS One., 4, e8407. 20027311 10.1371/journal.pone.0008407 PMC2793427

[27] Simpson J.T. Wong K. Jackman S.D. (2009) ABySS: a parallel assembler for short read sequence data. Genome Res., 19, 1117–1123. 19251739 10.1101/gr.089532.108 PMC2694472

[28] Chang X. Wang K. (2012) wANNOVAR: annotating genetic variants for personal genomes via the web. J. Med. Genet., 10, 1136/100918. 10.1136/jmedgenet-2012-100918 PMC3556337 22717648

[29] Wang K. Li M. Hakonarson H. (2010) ANNOVAR: functional annotation of genetic variants from high-throughput sequencing data. Nucleic Acids Res., 38, e164. 20601685 10.1093/nar/gkq603 PMC2938201

[30] Ge D. Ruzzo E.K. Shianna K.V. (2011) SVA: software for annotating and visualizing sequenced human genomes. Bioinformatics, 27, 1998–2000. 21624899 10.1093/bioinformatics/btr317 PMC3129530

[31] Lee E. Helt GA. Reese JT. (2013) Web Apollo: a web-based genomic annotation editing platform. Genome Biol., 14, R93. 24000942 10.1186/gb-2013-14-8-r93 PMC4053811

[32] Sana M.E. Iascone M. Marchetti D. (2011) GAMES identifies and annotates mutations in next-generation sequencing projects. Bioinformatics, 27, 9–13. 20971986 10.1093/bioinformatics/btq603

[33] Crisan A. Goya R. Ha G. (2012) Mutation discovery in regions of segmental cancer genome amplifications with CoNAn-SNV: a mixture model for next generation sequencing of tumors. PLoS One., 7, e41551. 22916110 10.1371/journal.pone.0041551 PMC3420914

[34] Wilm A. Aw P.P. Bertrand D. (2012) LoFreq: a sequence-quality aware, ultra-sensitive variant caller for uncovering cell-population heterogeneity from high-throughput sequencing datasets. Nucleic Acids Res., 40, 11189–11201. 23066108 10.1093/nar/gks918 PMC3526318

[35] DePristo M.A. Banks E. Poplin R. (2011) A framework for variation discovery and genotyping using next-generation DNA sequencing data. Nat. Genet., 43, 491–498. 21478889 10.1038/ng.806 PMC3083463

[36] Roth A. Ding J. Morin R. (2012) JointSNVMix: a probabilistic model for accurate detection of somatic mutations in normal/tumour paired next-generation sequencing data. Bioinformatics, 28, 907–913. 22285562 10.1093/bioinformatics/bts053 PMC3315723

[37] Li H. Handsaker B. Wysoker A. (2009) The sequence alignment/map format and SAMtools. Bioinformatics, 25, 2078–2079. 19505943 10.1093/bioinformatics/btp352 PMC2723002

[38] Goya R. Sun M.G. Morin R.D. (2010) SNVMix: predicting single nucleotide variants from next-generation sequencing of tumors. Bioinformatics, 26, 730–736. 20130035 10.1093/bioinformatics/btq040 PMC2832826

[39] Saunders C.T. Wong W.S. Swamy S. (2012) Strelka: accurate somatic small-variant calling from sequenced tumor-normal sample pairs. Bioinformatics, 28, 1811–1817. 22581179 10.1093/bioinformatics/bts271

[40] Larson D.E. Harris C.C. Chen K. (2012) SomaticSniper: identification of somatic point mutations in whole genome sequencing data. Bioinformatics, 28, 311–317. 22155872 10.1093/bioinformatics/btr665 PMC3268238

[41] Koboldt D.C. Chen K. Wylie T. (2009) VarScan: variant detection in massively parallel sequencing of individual and pooled samples. Bioinformatics, 25, 2283–2285. 19542151 10.1093/bioinformatics/btp373 PMC2734323

[42] Albers C.A. Lunter G. MacArthur D.G. (2011) Dindel: accurate indel calls from short-read data. Genome Res., 6, 961–973. 20980555 10.1101/gr.112326.110 PMC3106329

[43] Ye K. Schulz M.H. Long Q. (2009) Pindel: a pattern growth approach to detect break points of large deletions and medium sized insertions from paired-end short reads. Bioinformatics, 25, 2865–2871. 19561018 10.1093/bioinformatics/btp394 PMC2781750

[44] Lee S. Hormozdiari F. Alkan C. (2009) MoDIL: detecting small indels from clone-end sequencing with mixtures of distributions. Nat. Methods, 6, 473–474. 19483690 10.1038/nmeth.f.256

[45] Zeng F. Jiang R. Chen T. (2013) PyroHMMvar: a sensitive and accurate method to call short indels and SNPs for ion torrent and 454 data. Bioinformatics, 29, 2859–2868. 23995392 10.1093/bioinformatics/btt512 PMC3888126

[46] Zhang J. Wang J. Wu Y. (2012) An improved approach for accurate and efficient calling of structural variations with low-coverage sequence data. BMC Bioinformatics, 13, S6. 10.1186/1471-2105-13-S6-S6 PMC3358659 22537045

[47] Fan X. Abbott T.E. Larson D. (2014) Breakdancer - identification of genomic structural variation from paired-end read mapping. Curr. Protoc. Bioinformatics, 45, 15.6.1–15.6.11. 10.1002/0471250953.bi1506s45 PMC4138716 25152801

[48] Kim M. Farnoud F. Milenkovic O. (2015) HyDRA: gene prioritization via hybrid distance-score rank aggregation. Bioinformatics, 31:1034–43. 25411330 10.1093/bioinformatics/btu766

[49] Korbel J.O. Abyzov A. Mu X.J. (2009) PEMer: a computational framework with simulation-based error models for inferring genomic structural variants from massive paired-end sequencing data. Genome Biol., 10, R23. 19236709 10.1186/gb-2009-10-2-r23 PMC2688268

[50] Klein H.U. Bartenhagen C. Kohlmann A. (2011) R453Plus1Toolbox: an R/Bioconductor package for analyzing Roche 454 Sequencing data. Bioinformatics, 27, 1162–1163. 21349869 10.1093/bioinformatics/btr102

[51] Wong K. Keane T.M. Stalker J. (2010) Enhanced structural variant and breakpoint detection using SVMerge by integration of multiple detection methods and local assembly. Genome Biol., 11, R128. 21194472 10.1186/gb-2010-11-12-r128 PMC3046488

[52] Zeitouni B. Boeva V. Janoueix-Lerosey I. (2010) SVDetect: a tool to identify genomic structural variations from paired-end and mate-pair sequencing data. Bioinformatics, 26, 1895–1896. 20639544 10.1093/bioinformatics/btq293 PMC2905550

[53] Hormozdiari F. Hajirasouliha I. Dao P. (2010) Next-generation VariationHunter: combinatorial algorithms for transposon insertion discovery. Bioinformatics, 26, i350–i357. 20529927 10.1093/bioinformatics/btq216 PMC2881400

[54] Zhang Q. Ding L. Larson D.E. (2010) CMDS: a population-based method for identifying recurrent DNA copy number aberrations in cancer from high-resolution data. Bioinformatics, 26, 464–469. 20031968 10.1093/bioinformatics/btp708 PMC2852218

[55] Ivakhno S. Royce T. Cox A.J. (2010) CNAseg–a novel framework for identification of copy number changes in cancer from second-generation sequencing data. Bioinformatics, 26, 3051–3058. 20966003 10.1093/bioinformatics/btq587

[56] Abyzov A. Urban A.E. Snyder M. (2011) CNVnator: an approach to discover, genotype, and characterize typical and atypical CNVs from family and population genome sequencing. Genome Res., 21, 974–984. 21324876 10.1101/gr.114876.110 PMC3106330

[57] Boeva V. Zinovyev A. Bleakley K. (2011) Control-free calling of copy number alterations in deep-sequencing data using GC-content normalization. Bioinformatics, 27, 268–269. 21081509 10.1093/bioinformatics/btq635 PMC3018818

[58] Yoon S. Xuan Z. Makarov V. (2009) Sensitive and accurate detection of copy number variants using read depth of coverage. Genome Res., 9, 1586–1592. 19657104 10.1101/gr.092981.109 PMC2752127

[59] Chiang D.Y. Getz G. Jaffe D.B. (2009) High-resolution mapping of copy-number alterations with massively parallel sequencing. Nat. Methods, 6, 99–103. 19043412 10.1038/nmeth.1276 PMC2630795

[60] Xie C. Tammi M.T. (2009) CNV- seq, a new method to detect copy number variation using high-throughput sequencing. BMC Bioinformatics, 10, 80. 19267900 10.1186/1471-2105-10-80 PMC2667514

[61] Alkan C. Kidd J.M. Marques-Bonet T. (2009) Personalized copy number and segmental duplication maps using next-generation sequencing. Nat. Genet., 41, 1061–1067. 19718026 10.1038/ng.437 PMC2875196

[62] Adzhubei I.A. Schmidt S. Peshkin L. (2010) A method and server for predicting damaging missense mutations. Nat. Methods, 7, 248–249. 20354512 10.1038/nmeth0410-248 PMC2855889

[63] Carter H. Chen S. Isik L. (2009) Cancer-specific high-throughput annotation of somatic mutations: computational prediction of driver missense mutations. Cancer Res., 69, 6660–6667. 19654296 10.1158/0008-5472.CAN-09-1133 PMC2763410

[64] Sim N.L. Kumar P. Hu J. (2012) SIFT web server: predicting effects of amino acid substitutions on proteins. Nucleic Acids Res., 40, W452– W457. 22689647 10.1093/nar/gks539 PMC3394338

[65] http://www.genomenewsnetwork.org/resources/whats_a_genome/Chp1_4_1.shtml

[66] Trapnell C. Pachter L. Salzberg S.L. (2009) TopHat: discovering splice junctions with RNA-Seq. Bioinformatics, 25, 1105–1111. 19289445 10.1093/bioinformatics/btp120 PMC2672628

[67] De Bona F. Ossowski S. Schneeberger K. (2008) Optimal spliced alignments of short sequence reads. Bioinformatics, 24, i174–i180. 18689821 10.1093/bioinformatics/btn300

[68] Wang K. Singh D. Zeng Z. (2010) MapSplice: accurate mapping of RNA-seq reads for splice junction discovery. Nucleic Acids Res., 38, e178. 20802226 10.1093/nar/gkq622 PMC2952873

[69] Au K.F. Jiang H. Lin L. (2010) Detection of splice junctions from paired-end RNA-seq data by SpliceMap. Nucleic Acids Res., 38, 4570–4578. 20371516 10.1093/nar/gkq211 PMC2919714

[70] Dobin A. Davis C.A. Schlesinger F. (2013) STAR: ultrafast universal RNA-seq aligner. Bioinformatics, 29, 15–21. 23104886 10.1093/bioinformatics/bts635 PMC3530905

[71] Huang S. Zhang J. Li R. (2011) SOAPsplice: genome-wide ab initio detection of splice junctions from RNA-seq data. Front. Genet., 2, 46. 22303342 10.3389/fgene.2011.00046 PMC3268599

[72] Bryant D.W. Jr Shen R. Priest H.D. (2010) Supersplat - spliced RNA-seq alignment. Bioinformatics, 26, 1500–1505. 20410051 10.1093/bioinformatics/btq206 PMC2881391

[73] Robinson M.D. McCarthy D.J. Smyth G.K. (2010) edgeR: a Bioconductor package for differential expression analysis of digital gene expression data. Bioinformatics, 26, 139–140. 19910308 10.1093/bioinformatics/btp616 PMC2796818

[74] Trapnell C. Hendrickson D.G. Sauvageau M. (2010) Differential analysis of gene regulation at transcript resolution with RNA-seq. Nat. Biotechnol., 31, 46–53. 23222703 10.1038/nbt.2450 PMC3869392

[75] Anders S. Huber W. (2010) Differential expression analysis for sequence count data. Genome Biol., 11, R106. 20979621 10.1186/gb-2010-11-10-r106 PMC3218662

[76] Langmead B. Hansen K.D. Leek J.T. (2010) Cloud-scale RNA-sequencing differential expression analysis with Myrna. Genome Biol., 11, R83. 20701754 10.1186/gb-2010-11-8-r83 PMC2945785

[77] Katz Y. Wang E.T. Airoldi E.M. (2010) Analysis and design of RNAsequencing experiments for identifying isoform regulation. Nat. Methods, 7, 1009–1015. 21057496 10.1038/nmeth.1528 PMC3037023

[78] Anders S. Reyes A. Huber W. (2012) Detecting differential usage of exons from RNA-seq data. Genome Res., 10, 2008–2017. 22722343 10.1101/gr.133744.111 PMC3460195

[79] Griffith M. Griffith O.L. Mwenifumbo J. (2010) Alternative expression analysis by RNA sequencing. Nat. Methods, 7, 843–847. 20835245 10.1038/nmeth.1503

[80] Xie Y. Wu G. Tang J. (2014) SOAPdenovo-Trans: de novo transcriptome assembly with short RNA-Seq reads. Bioinformatics, 30, 1660–1666. 24532719 10.1093/bioinformatics/btu077

[81] McPherson A. Hormozdiari F. Zayed A. (2011) deFuse: an algorithm for gene fusion discovery in tumor RNA-Seq data. PLoS Comput. Biol., 7, e1001138. 21625565 10.1371/journal.pcbi.1001138 PMC3098195

[82] Piazza R. Pirola A. Spinelli R. (2012) FusionAnalyser: a new graphical, event-driven tool for fusion rearrangements discovery. Nucleic Acids Res., 40, e123. 22570408 10.1093/nar/gks394 PMC3439881

[83] Li Y. Chien J. Smith D.I. (2011) FusionHunter: identifying fusion transcripts in cancer using paired-end RNA-seq. Bioinformatics, 27, 1708–1710. 21546395 10.1093/bioinformatics/btr265

[84] Ge H. Liu K. Juan T. (2011) FusionMap: detecting fusion genes from next-generation sequencing data at base- pair resolution. Bioinformatics, 27, 1922–1928. 21593131 10.1093/bioinformatics/btr310

[85] Sboner A. Habegger L. Pflueger D. (2010) FusionSeq: a modular framework for finding gene fusions by analyzing paired-end RNA-sequencing data. Genome Biol., 11, R104. 20964841 10.1186/gb-2010-11-10-r104 PMC3218660

[86] Wu J. Zhang W. Huang S. (2013) SOAPfusion: a robust and effective computational fusion discovery tool for RNA-seq reads. Bioinformatics, 29, 2971–2978. 24123671 10.1093/bioinformatics/btt522

[87] Kim D. Salzberg S.L. (2011) TopHat-Fusion: an algorithm for discovery of novel fusion transcripts. Genome Biol., 12, R72. 21835007 10.1186/gb-2011-12-8-r72 PMC3245612

[88] Rhodes D.R. Chinnaiyan A.M. (2005) Integrative analysis of the cancer transcriptome. Nat. Genet., 37, S31–S37. 15920528 10.1038/ng1570

[89] Patel R.K. Jain M. (2012) NGS QC Toolkit: a toolkit for quality control of next generation sequencing data. PLoS One, 7, e30619. 22312429 10.1371/journal.pone.0030619 PMC3270013

[90] Schröder J. Schröder H. Puglisi S.J. (2009) SHREC: a short-read error correction method. Bioinformatics, 25, 2157–2163. 19542152 10.1093/bioinformatics/btp379

[91] Lassmann T. Hayashizaki Y. Daub C.O. (2009) TagDust - a program to eliminate artifacts from next generation sequencing data. Bioinformatics, 25, 2839–2840. 19737799 10.1093/bioinformatics/btp527 PMC2781754

[92] Kao W.C. Stevens K. Song Y.S. (2009) BayesCall: a model-based basecalling algorithm for high-throughput short-read sequencing. Genome Res., 19, 1884–1895. 19661376 10.1101/gr.095299.109 PMC2765266

[93] Kircher M. Stenzel U. Kelso J. (2009) Improved base calling for the Illumina Genome Analyzer using machine learning strategies. Genome Biol., 10, R83. 19682367 10.1186/gb-2009-10-8-r83 PMC2745764

[94] Whiteford N. Skelly T. Curtis C. (2009) Swift: primary data analysis for the Illumina Solexa sequencing platform. Bioinformatics, 25, 2194–2199. 19549630 10.1093/bioinformatics/btp383 PMC2734321

[95] Ilie L. Fazayeli F. Ilie S. (2011) HiTEC: accurate error correction in high-throughput sequencing data. Bioinformatics, 27, 295–302. 21115437 10.1093/bioinformatics/btq653

[96] Liu Y. Schröder J. Schmidt B. (2013) A multistage k-mer spectrum-based error corrector for Illumina sequence data. Bioinformatics, 29, 308–315. 23202746 10.1093/bioinformatics/bts690

[97] Kao W.C. Chan A.H. Song Y.S. (2011) ECHO: a reference-free short-read error correction algorithm. Genome Res., 21, 1181–1192. 21482625 10.1101/gr.111351.110 PMC3129260

[98] Lim E.C. Müller J. Hagmann J. (2014) Trowel: a fast and accurate error correction module for Illumina sequencing reads. Bioinformatics, 30, 3264–3265. 25075116 10.1093/bioinformatics/btu513

[99] Yang X. Dorman K.S. Aluru S. (2010) Reptile: representative tiling for short read error correction. Bioinformatics, 26, 2526–2533. 20834037 10.1093/bioinformatics/btq468

[100] Wirawan A. Harris R.S. Liu Y. (2014) HECTOR: a parallel multistage homopolymer spectrum based error corrector for 454 sequencing data. BMC Bioinformatics,15, 131. 24885381 10.1186/1471-2105-15-131 PMC4023493

[101] Liu Y. Schmidt B. Maskell D.L. (2011) DecGPU: distributed error correction on massively parallel graphics processing units using CUDA and MPI. BMC Bioinformatics, 12, 85. 21447171 10.1186/1471-2105-12-85 PMC3072957

[102] Yang X. Liu D. Liu F. (2013) HTQC: a fast quality control toolkit for Illumina sequencing data. BMC Bioinformatics, 14, 33. 23363224 10.1186/1471-2105-14-33 PMC3571943

[103] Zhou Q. Su X. Wang A. (2013) QC-Chain: fast and holistic quality control method for next-generation sequencing data. PLoS One, 8, e60234. 23565205 10.1371/journal.pone.0060234 PMC3615005

[104] Davis M.P van.Dongen S. Abreu-Goodger C. (2013) Kraken: a set of tools for quality control and analysis of high-throughput sequence data. Methods, 63, 41–49. 23816787 10.1016/j.ymeth.2013.06.027 PMC3991327

[105] Plass C. (2002) Cancer epigenomics, Hum. Mol. Genet., 11, 2479–2488. 12351584 10.1093/hmg/11.20.2479

[106] Zhang Y. Liu T. Meyer C.A. (2008) Model-based analysis of ChIP-Seq (MACS). Genome Biol., 9, R137. 18798982 10.1186/gb-2008-9-9-r137 PMC2592715

[107] Rozowsky J. Euskirchen G. Auerbach R.K. (2009) PeakSeq enables systematic scoring of ChIP-seq experiments relative to controls. Nat. Biotechnol., 27, 66–75. 19122651 10.1038/nbt.1518 PMC2924752

[108] Zytnicki M. Quesneville H. (2011) S-MART, a software toolbox to aid RNA- Seq data analysis. PLoS One., 6, e25988. 21998740 10.1371/journal.pone.0025988 PMC3188586

[109] Xu S. Grullon S. Ge K. (2014) Spatial clustering for identification of ChIP-enriched regions (SICER) to map regions of histone methylation patterns in embryonic stem cells. Methods Mol. Biol., 1150, 97–111. 24743992 10.1007/978-1-4939-0512-6_5 PMC4152844

[110] Timothy L. Bailey Bodén M. (2009) MEME SUITE: tools for motif discovery and searching. Nucleic Acids Res., 37, W202–W208. 19458158 10.1093/nar/gkp335 PMC2703892

[111] Guo Y. Mahony S. Gifford D.K. (2012) High resolution genome wide binding event finding and motif discovery reveals transcription factor spatial binding constraints. PLoS Comput. Biol., 8, e1002638. 22912568 10.1371/journal.pcbi.1002638 PMC3415389

[112] Bailey T.L. (2011) DREME: motif discovery in transcription factor ChIP-seq data. Bioinformatics, 27, 1653–1659. 21543442 10.1093/bioinformatics/btr261 PMC3106199

[113] Liu Y. Siegmund K.D. Laird P.W. (2012) Bis-SNP: combined DNA methylation and SNP calling for Bisulfite-seq data. Genome Biol., 13, R61. 22784381 10.1186/gb-2012-13-7-r61 PMC3491382

[114] Xi Y. Li W. (2009) BSMAP: whole genome bisulfite sequence mapping program. BMC Bioinformatics, 10, 232. 19635165 10.1186/1471-2105-10-232 PMC2724425

[115] Harris E.Y. Ponts N. Levchuk A. (2009) BRAT: bisulfite-treated reads analysis tool. Bioinformatics, 26, 2499. 10.1093/bioinformatics/btp706 PMC3716225 20031974

[116] Kreck B. Marnellos G. Richter J. (2012) B-SOLANA: an approach for the analysis of two-base encoding bisulfite sequencing data. Bioinformatics, 28, 428–429. 22155865 10.1093/bioinformatics/btr660 PMC3268236

[117] Campagna D. Telatin A. Forcato C. (2013) PASS-bis: a bisulfite aligner suitable for whole methylome analysis of Illumina and SOLiD reads. Bioinformatics, 29, 268–270. 23162053 10.1093/bioinformatics/bts675

[118] Krueger F. Andrews S.R. (2011) Bismark: a flexible aligner and methylation caller for Bisulfite-Seq applications. Bioinformatics, 27, 1571–1572. 21493656 10.1093/bioinformatics/btr167 PMC3102221

[119] Gruntman E. Qi Y. Slotkin R.K. (2008) Kismeth: analyzer of plant methylation states through bisulfite sequencing. BMC Bioinformatics, 9, 371. 18786255 10.1186/1471-2105-9-371 PMC2553349

[120] Chen P.Y. Cokus S.J. Pellegrini M. (2010) BS Seeker: precise mapping for bisulfite sequencing, BMC Bioinformatics, 11, 203. 20416082 10.1186/1471-2105-11-203 PMC2871274

[121] Akalin A. Kormaksson M. Li S. (2012) methylKit: a comprehensive R package for the analysis of genome-wide DNA methylation profiles. Genome Biol., 13, R87. 23034086 10.1186/gb-2012-13-10-r87 PMC3491415

[122] Pedersen B. Hsieh T.F. Ibarra C. (2011) MethylCoder: software pipeline for bisulfite-treated sequences. Bioinformatics., 27, 2435–2436. 21724594 10.1093/bioinformatics/btr394 PMC3157921

[123] Krzywinski M. Schein J. Birol I. (2009) Circos: an information aesthetic for comparative genomics. Genome Res., 19, 1639–1645. 19541911 10.1101/gr.092759.109 PMC2752132

[124] Robinson J.T. Thorvaldsdóttir H. Winckler W. (2011) Integrative genomics viewer. Nat. Biotechnol., 29, 24–26. 21221095 10.1038/nbt.1754 PMC3346182

[125] Thorvaldsdóttir H. Robinson J.T. Mesirov J.P. (2013) Integrative genomics viewer (IGV): high-performance genomics data visualization and exploration. Brief. Bioinform., 14, 178–192. 22517427 10.1093/bib/bbs017 PMC3603213

[126] Milne I. Stephen G. Bayer M. (2013) Using tablet for visual exploration of second-generation sequencing data. Brief. Bioinform., 14, 193–202. 22445902 10.1093/bib/bbs012

[127] Carver T. Böhme U. Otto T.D. (2010) BamView: viewing mapped read alignment data in the context of the reference sequence. Bioinformatics, 26, 676–677. 20071372 10.1093/bioinformatics/btq010 PMC2828118

[128] Carver T. Harris S.R. Otto T.D. (2012) BamView: visualizing and interpretation of next-generation sequencing read alignments. Brief. Bioinform. 14, 203–212. 22253280 10.1093/bib/bbr073 PMC3603209

[129] Huang W. Marth G. (2008) EagleView: a genome assembly viewer for next-generation sequencing technologies. Genome Res., 18, 1538–1543. 18550804 10.1101/gr.076067.108 PMC2527701

[130] Arner E. Hayashizaki Y. Daub C.O. (2010) NGSView: an extensible open source editor for next-generation sequencing data. Bioinformatics., 26, 125–126. 19855106 10.1093/bioinformatics/btp611 PMC2796816

[131] Zhang Z. Lin H. Ma B. (2010) ZOOM Lite: next-generation sequencing data mapping and visualization software. Nucleic Acids Res., 38, W743–W748. 20530531 10.1093/nar/gkq538 PMC2896157

[132] Goldman M. Craft B. Swatloski T. (2015) the ucsc cancer genomics browser: update 2015. Nucleic Acids Res., 43, D670–D681. 25392408 10.1093/nar/gku1073 PMC4383911

[133] Lajugie J. Bouhassira E.E. (2011) GenPlay, a multipurpose genome analyzer and browser. Bioinformatics, 27, 1889–1893. 21596789 10.1093/bioinformatics/btr309

[134] Lajugie J. Fourel N. Bouhassira E.E. (2015) GenPlay Multi-Genome, a tool to compare and analyze multiple human genomes in a graphical interface. Bioinformatics, 1, 109–111. 25178461 10.1093/bioinformatics/btu588 PMC4402384

[135] Kong L. Wang J. Zhao S. (2012) ABrowse - a customizable next-generation genome browser framework. BMC Bioinformatics, 13, 2. 22222089 10.1186/1471-2105-13-2 PMC3265404

[136] Rutherford K. Parkhill J. Crook J. (2000) Artemis: sequence visualization and annotation. Bioinformatics, 16, 944–945. 11120685 10.1093/bioinformatics/16.10.944

[137] Carver T. Harris S.R. Berriman M. (2012) Artemis: an integrated platform for visualization and analysis of high-throughput sequence-based experimental data. Bioinformatics, 28, 464–469. 22199388 10.1093/bioinformatics/btr703 PMC3278759

[138] Nevado B. Perez-Enciso M. PIPELINER: a tool to evaluate NGS pipelines and optimize experimental designs for resequencing studies. EMBnet. J., 19.A, 64–65.

[139] Dai M. Thompson R. Maher. C. (2010) NGSQC: cross-platform quality analysis pipeline for deep sequencing data. BMC Genomics, 11, S7. 21143816 10.1186/1471-2164-11-S4-S7 PMC3005923

[140] Nagasaki H. Mochizuki T. Kodama Y. (2013) DDBJ read annotation pipeline: a cloud computing-based pipeline for high-throughput analysis of next-generation sequencing data. DNA Res. 20, 383–390. 23657089 10.1093/dnares/dst017 PMC3738164

[141] Torstenson E.S. Li B. Li C. (2013) ASAP: an environment for automated preprocessing of sequencing data. BMC Res. Notes, 6, 5. 23289815 10.1186/1756-0500-6-5 PMC3541347

[142] Chen K. Wallis J.W. Kandoth C. (2012) BreakFusion: targeted assembly-based identification of gene fusions in whole transcriptome paired-end sequencing data. Bioinformatics, 28, 1923–1924. 22563071 10.1093/bioinformatics/bts272 PMC3389765

[143] Morris T.J. Butcher L.M. Feber A. (2014) ChAMP: 450k chip analysis methylation pipeline. Bioinformatics, 30, 428–430. 24336642 10.1093/bioinformatics/btt684 PMC3904520

[144] Arumugam M. Harrington E.D. Foerstner K.U. (2010) SmashCommunity: a metagenomic annotation and analysis tool. Bioinformatics, 26, 2977–2978. 20959381 10.1093/bioinformatics/btq536

[145] Koren S. Treangen T.J. Hill C.M. (2014) Automated ensemble assembly and validation of microbial genomes. BMC Bioinformatics, 15, 126. 24884846 10.1186/1471-2105-15-126 PMC4030574

[146] Grant. G.R. Farkas M.H. Pizarro A.D. (2011) Comparative analysis of RNA-Seq alignment algorithms and the RNA-Seq unified mapper (RUM). Bioinformatics, 27, 2518–2528. 21775302 10.1093/bioinformatics/btr427 PMC3167048

[147] Klambauer G. Schwarzbauer K. Mayr A. (2012) cn.MOPS: mixture of Poissons for discovering copy number variations in next-generation sequencing data with a low false discovery rate. Nucleic Acids Res., 40, e69. 22302147 10.1093/nar/gks003 PMC3351174

[148] Andolfatto P. Davison D. Erezyilmaz D. (2011) Multiplexed shotgun genotyping for rapid and efficient genetic mapping. Genome Res., 21, 610–617. 21233398 10.1101/gr.115402.110 PMC3065708

[149] http://allaboutbioinfo.blogspot.in/2011/08/qseq-and-export-file-format-of-illumina.html

[150] http://blog.goldenhelix.com/grudy/ngs-tools-and-formats-for-secondary-analysis-a-primer/

[151] https://tcga-data.nci.nih.gov/tcga/

[152] Sathya B. Akila P.D. Kumar G.R. (2014) NGS meta data analysis for identification of SNP and INDEL patterns in human airway transcriptome: a preliminary indicator for lung cancer. Appl. Transl. Genom., 4, 4–9. 10.1016/j.atg.2014.12.003 PMC4745382 26937342

[153] Shumway M. Cochrane G. Sugawara H. (2010) Archiving next generation sequencing data. Nucleic Acids Res., 38, D870–D871. 19965774 10.1093/nar/gkp1078 PMC2808927

